# Concordance between patient‐reported and physician‐documented comorbidities and symptoms among Stage 4 breast cancer patients

**DOI:** 10.1002/cam4.6632

**Published:** 2023-10-30

**Authors:** Saumya Umashankar, Amrita Basu, Laura Esserman, Laura van't Veer, Michelle E. Melisko

**Affiliations:** ^1^ University of California San Francisco California USA

**Keywords:** breast cancer, comorbidity, electronic health records, patient reported outcome measures, symptom assessment

## Abstract

**Background:**

Comorbidities and symptoms in metastatic breast cancer patients impact treatment decisions and influence prognosis and quality of life. The objective of this study is to examine the concordance between physician documentation and patient reports of comorbidities and symptoms to understand their comparative effectiveness.

**Methods:**

New patients with metastatic breast cancer completed an electronic intake survey assessing patient health history and symptoms. Physician documentation across 54 comorbidities and 42 symptoms was abstracted from notes for the corresponding clinic visits between November 2016 and March 2020. Concordance between patient reports and medical records for each condition and hazards ratios for each patient versus physician reported comorbidity and symptom were assessed.

**Results:**

A total of 168 patients were included in the analysis (age, median = 56 years, range = 29–86 years; 131 white [78.9%]). Twenty‐three of 54 comorbidities had a moderate to high level of agreement between patients and physicians (*κ* ≥ 0.40). Physicians documented higher numbers of comorbidities that can be objectively measured which also had higher concordance (e.g., diabetes [*κ* = 0.83] and hypertension [*κ* = 0.79]) while patients reported higher numbers of comorbidities that are more subjective which also had lower concordance (anxiety [*κ* = 0.30], GERD [*κ* = 0.36]). One physician‐documented and two patient‐reported comorbidities were significantly associated with survival (*p* < 0.05). Only 2 of 42 symptoms had a moderate to high level of agreement between patients and physicians. One physician‐documented and nine patient‐reported symptoms were significantly associated with decreased survival (*p* < 0.05).

**Conclusion:**

Agreement between patients' and physicians' reporting of comorbidities varies substantially, and patient reports can complement physician documentation. Physicians significantly underreported symptoms versus patients; thus, concordance was also low. Multiple patient‐reported symptoms were predictive of survival; thus, incorporating them can provide more informative estimates of predicted survival.

## 1 BACKGROUND

Comorbidities and symptoms in metastatic breast cancer patients can significantly influence treatment decisions, eligibility for clinical trials and influence prognosis and quality of life.^1^ With increasing use of an electronic medical record in which multiple providers are updating, and at times, copying forward patient health history, medications, and symptoms, there is often duplicity of data and erroneous entries carried forward.^2^ An issue that is worth study is whether physician documentation of comorbidities and symptoms or patient‐reported symptoms and health conditions more accurately represent a patient's true health history. Patient‐reported medical data offer a more cost and time‐efficient method of collecting a patient's health history^3^; however, its accuracy as compared to clinician data entered is uncertain. More health systems are also incorporating quality of life and symptom questionnaires as intake tools to streamline the clinical visit and screen for pain and distress. Further, patient‐reported outcomes are more commonly utilized in clinical trials to assess impact of treatment, but the physician reported adverse events and symptoms remain the gold standard. Similarly, clinician documentation of comorbidities and baseline and subsequent symptom burden, rather than patient report, is most often utilized to estimate patients' prognosis and choices of oncology treatment.

This study aims to fill the gap in literature by evaluating the concordance between patient reports and physician documentation of comorbidities and symptoms in an electronic medical record to (1) reliably document breast cancer patients' health histories, (2) identify comorbidities and symptoms that may be more comprehensively reported by patients rather than physicians, and (3) understand whether patient‐reported or physician‐documented comorbidities or symptoms are more predictive of patient survival.

## 2 METHODS

### 2.1 Data collection and outcome measures

**FIGURE 1:**
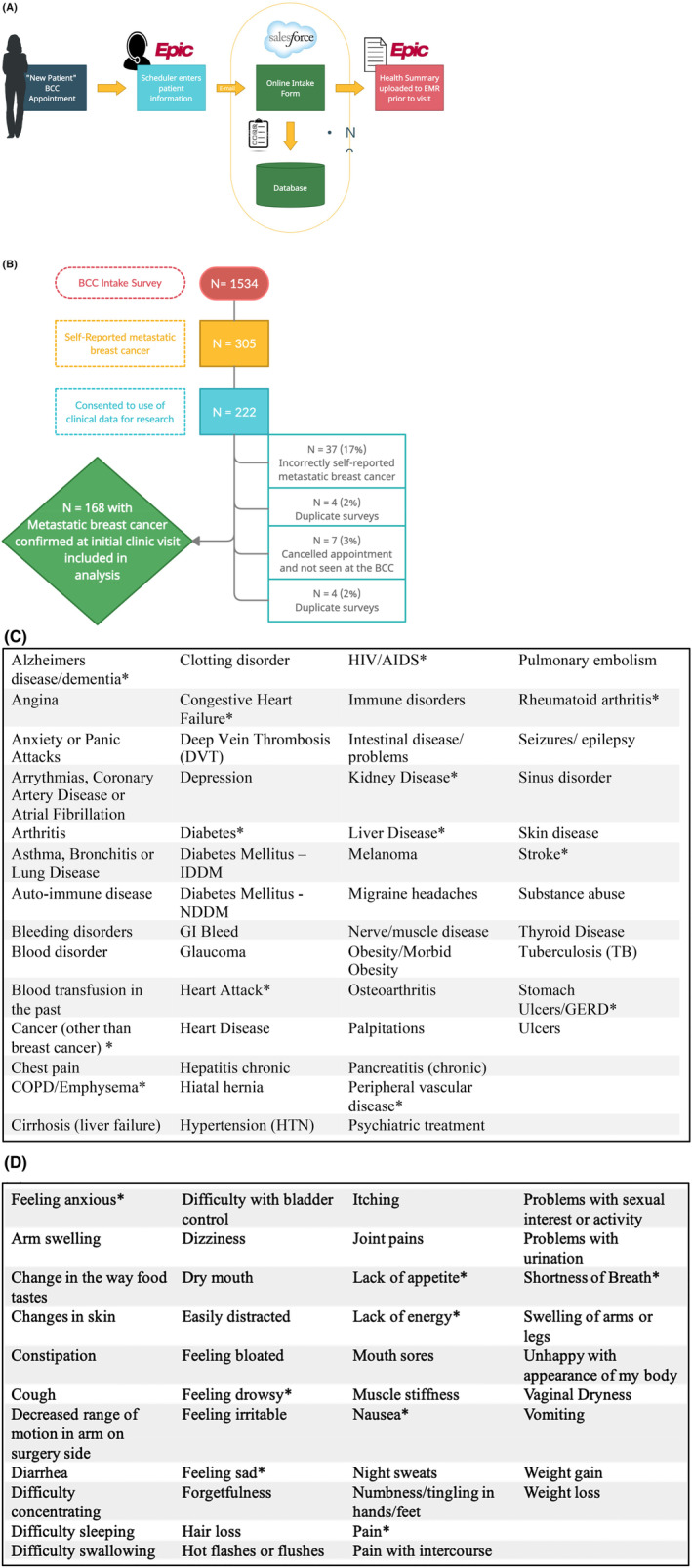
Methods. (A) UCSF's BCC electronic intake survey process; (B) Methodology of obtaining study population; (C) List of comorbidities assessed. Asterisks (*) represent comorbidities that overlap with the Charlson Comorbidity Index (CCI); (D) List of symptoms assessed. Asterisks (*) represent symptoms that overlap with the Edmonton Symptom Assessment Scale (ESAS).

All patients coming to UCSF's Breast Care Center (BCC) as a new patient are administered an electronic intake survey (Figure 1A) that includes patient‐reported health history, comorbidities, and symptoms in addition to demographic data. Patients complete this survey as a part of their routine clinical care; however, patients can consent to use of their survey data and clinical information for research purposes. The primary purpose of the intake survey of patient‐reported comorbities and symptom was to trigger referrals to supportive care services including psycho‐oncology, the behavioral sleep clinic, social work, nutritional counseling, peer support, oncofertility, genetic counseling, and smoking cessation. The summary data that are recorded in the electronic medical record contain summary PROMIS scores and responses to trigger questions that generated automatic referrals to supportive care services. The review and use of the patient‐reported survey data in real time by clinicians in the BCC is highly variably, with only a few providers reviewing the summary of survey responses before or during a clinic visit, while most providers do not use the summary report of patient entered data at all to document and patient's health conditions or symptoms. Independently, as standard of care in a clinical encounter, the physicians document patients' comorbidities and symptoms as directly ascertained during the appointment through verbal questioning of the patient and review of the patient's prior medical records.

Between November 2016 and March 2020, 305 patients self‐reported metastatic breast cancer (described as “breast cancer spread to sites other than breast or axillary lymph nodes”) prior to their initial BCC clinic visit, and 222 consented to use of their clinical data and survey responses for research (Figure 1B).

The surveys asked patients to report on the presence of 54 comorbidities (“Has a doctor ever told you that you have any of the following health conditions?,” listed in Figure 1C) and presence of 42 symptoms in the past week (“During the past week, have you had any of the following symptoms?,” listed in Figure 1D). Independently, the data were documented as a part of the physician's clinical note of the patients' initial clinic visit was retrospectively reviewed to ascertain physician‐documented comorbidities and symptoms. Comorbidities were defined as conditions that were documented in the past medical history, disease specific problem list, or as a diagnosis in the assessment and plan portion of the clinic note. Symptoms were defined as any symptom included in the clinical note narrative, review of systems or as an item in the assessment and plan portion of the clinic note. Patient and physician documents were summarized for 54 comorbidities (Figure 1C) and 42 symptoms (Figure 1D). The comorbidities assessed in the survey included all the comorbidities in the most commonly used comorbidity indices, including the Charlson Comorbidity Index,^4^ Elixhauser Index,^5^ Chronic Disease Score,^6^ and the Health‐related Quality of Life Comorbidity Index.^7^ The symptoms assessed in the survey are included in the most commonly used symptom assessment banks in breast cancer, including the PRO‐CTCAE^8^ and the Edmonton Symptom Assessment Scale (ESAS).^9^

### 2.2 Statistical analyses

We describe the presence of each comorbidity and symptom based on patient report, physician documentation, both or neither. Concordance between patient and physician reports of comorbidities and symptoms was quantified using Cohen's kappa (*κ*). *κ* reports the agreement between two information sources by providing a quantitative measurement of interobserver agreement, accounting for chance agreement. *κ* is standardized to a scale of −1 to 1, where 0 represents expected agreement by chance alone, −1 is perfect disagreement, and 1 is perfect agreement between the two sources.^10^ Level of agreement was classified using Landis and Koch thresholds with agreement as poor or slight (*κ* < 0.20), fair (*κ* ≥0.20 to <0.40), moderate (*κ* ≥0.40 to <0.60), substantial (*κ* ≥0.60 to <0.80), or almost perfect (*κ* ≥ 0.80).^11^ We performed subgroup logistic regression analyses based on age, race, educational level, and site of metastatic disease to assess covariates associated with agreement between patient reports and physician documents, with separate models for each comorbidity and symptoms assessed.

Cox‐proportional hazards models were used to determine hazard ratios (HR) for survival and corresponding 95% confidence intervals associated with each patient‐reported, physician‐documented, and concordant comorbidity and symptoms, after controlling for clinical covariates, such as age, site of metastatic disease (presence of metastases to brain, liver, lung, bone, or other), hormone receptor status, HER2 status, time since diagnosis of metastatic disease and line of therapy at initial clinic visit. Separate cox‐proportional hazards models were generated to develop hazards associated with each patient‐reported and physician‐documented comorbidity with greater than five reports and each patient reported and physician‐symptom with greater than 10 reports, using a binary outcome of death and continuous measure of time to event (death or last‐follow‐up).

All statistical analyses were performed using R and RStudio, version 3.6.3 and 1.2.5033 respectively.

### 2.3 Ethics approval

Approval was obtained from the UCSF ethics committee. The procedures used in this study adhere to the tenets of the Declaration of Helsinki. Informed consent was obtained from all individual participants included in the study. Patients provided written informed consent for publishing their deidentified data.

## 3 RESULTS

### 3.1 Study population

Of the 222 patients that were included in the study, 37 patients (17%) incorrectly reported having metastatic breast cancer and 16 (7%) were ineligible (Figure 1B). Thus, 168 patients with confirmed metastatic disease were included in the analysis (median age, 56 years; age range, 29–86 years; median time from diagnosis of metastatic breast cancer, 0.46 years). This cohort was demographically diverse, with 131 white participants (78.9%) and 42 non‐white participants (25.0%),* 53 (31.6%) with some college or less, 110 (65.1%) were not employed (either full or part‐time). Clinical and demographic characteristics of the patient population is summarized in Table 1.

**TABLE 1:** Clinical and demographic characteristics of study population.

Description	Study population
*N*	168
Age	
Mean (SD)	56.44 (12.02)
Median	55.90
Range	29–86
Race^a^	
Black or African American	10 (6.0%)
White	131 (78.9%)
Asian	21 (12.7%)
Other	11 (6.6%)
Prefer not to answer or don't know	5 (3.0%)
Hispanic	
Yes	14 (8.4%)
No	149 (89.8%)
Prefer not to say	3 (1.8%)
Marital status	
Divorced	16 (9.6%)
Living with a partner in a marriage‐like relationship	12 (7.2%)
Married	118 (71.1%)
Never married	7 (4.2%)
Separated	8 (4.8%)
Widowed	5 (3.0%)
Education	
Some high school or less	5 (3.0%)
High school graduate	9 (5.4%)
Some college or technical school	39 (23.5%)
College graduate or more	113 (68.1%)
Employment	
Employed full‐time (including self‐employed)	37 (22.3%)
Employed part‐time (including self‐employed)	19 (11.4%)
On leave of absence	27 (16.3%)
Retired (not due to ill health)	42 (25.3%)
Disabled and/or retired because of ill health	29 (17.5%)
Full‐time homemaker	9 (5.4%)
Unemployed	10 (6%)
Student	1 (0.6%)
Other	9 (5.4%)
Time from diagnosis of metastatic breast cancer to survey completion	
Mean (SD)	1.45 (2.19)
Median	0.46
Range	0.01–12.42
Line of therapy for metastatic disease	
Mean (SD)	1.67 (1.68)
Median	1
Range	0–8
HER2 status	
Negative	136 (81.0%)
Positive	32 (19.0%)
HR status	
Negative	44 (26.2%)
Positive	124 (73.8%)
Site of metastases	
Brain	19 (11.3%)
Liver	56 (33.3%)
Bone	95 (56.5%)
Lung	57 (33.9%)
Other	46 (27.7%)
Mortality (from time of survey completion)	
At 6‐month follow‐up from survey completion	22 of 166 (13.3%)
At 1‐year follow‐up from survey completion	42 of 146 (28.8%)
At 2‐year follow‐up from survey completion	63 of 116 (54.3%)
At 3‐year follow‐up from survey completion	73 of 89 (82.0%)
Mortality (from time of diagnosis of metastatic breast cancer)	
At 6‐month follow‐up from metastatic diagnosis	9 of 166 (5.4%)
At 1‐year follow‐up from metastatic diagnosis	15 of 162 (9.3%)
At 2‐year follow‐up from metastatic diagnosis	35 of 141 (24.8%)
At 3‐year follow‐up from metastatic diagnosis	52 of 113 (46.0%)

^a^ Race was a multiple‐option checkbox, and patients could select more than one of the options to best describe their self‐identified race.

### 3.2 Comorbidity analyses

**FIGURE 2:**
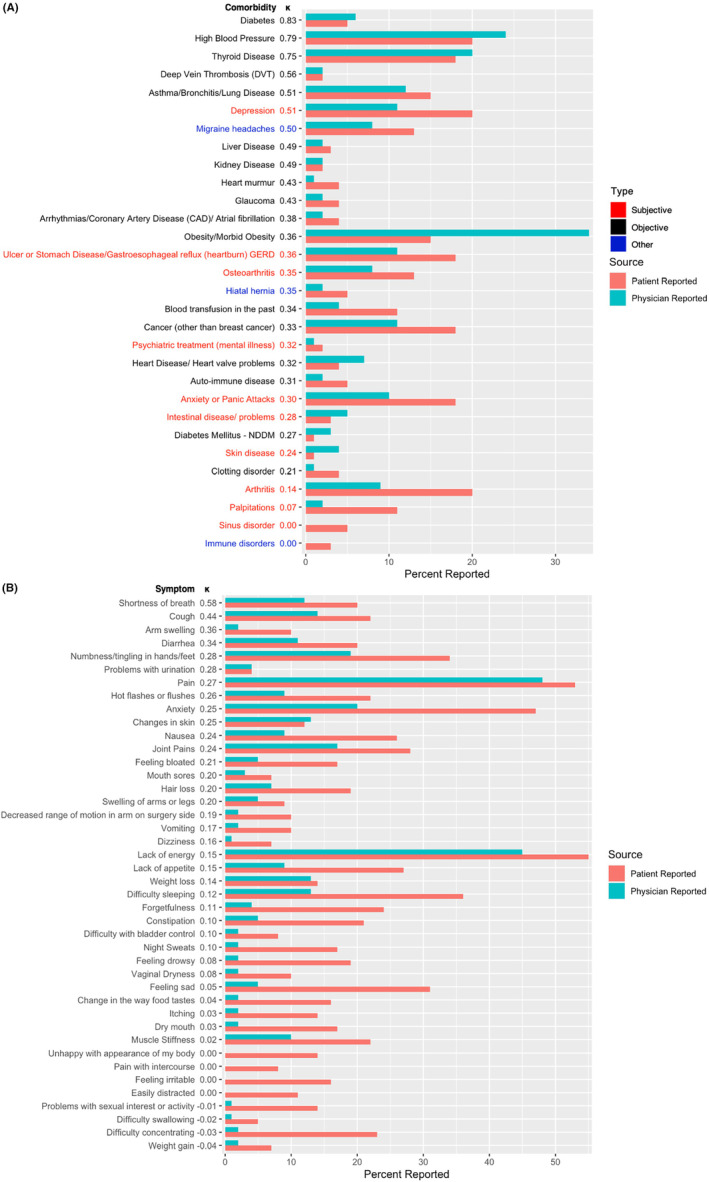
Concordance between patient‐reported and physician‐documented comorbidities (A) and symptoms (B). Comorbidities (in A) and symptoms (in B) are ordered by *κ*, and the *x*‐axis represents the percent reports by patients and physicians.

Patient and physician reporting of each comorbidity, level of agreement, and κ are summarized in Figure 2A and Table S1. Figure 2A reports on the concordance between patient and physician documentation of comorbidities. Objective comorbidities are those that can be objectively measured through a diagnostic test (laboratory tests, imaging, other tests, etc.). Subjective comorbidities represent the other comorbidities that cannot be measured directly through diagnostic testing. These comorbidities are usually established based assessment of symptoms or through subjective clinical or physical examination. The average Charlson Comorbidity Index for this population was 6.4 ± 0.88 (range 6–11), with 6 being the baseline score for patients with no other comorbidity other than their metastatic breast cancer. Highest comorbidities documented by physicians were obesity, hypertension, and thyroid disease while highest comorbidities reported by patients were hypertension, depression, and arthritis. Twenty‐three of 54 comorbidities had a moderate to high level of agreement between patients and physicians (*κ* ≥ 0.40). Agreement was highest for diabetes, hypertension, and thyroid disease. Agreement was moderate for asthma/bronchitis/lung disease and depression and low for anxiety, arthritis, obesity, and stomach ulcers/GERD.

There was substantial variance in the concordance of comorbidities and symptoms reported by patients versus physicians. Overall, concordance was higher for comorbidities than symptoms. Physicians documented higher numbers of comorbidities that can be objectively measured (through laboratory values, imaging, tests, etc.; e.g., diabetes or hypertension) while patients reported higher numbers of comorbidities that are more subjective (anxiety, GERD). Further, concordance was also higher for comorbidities that can be objectively assessed than for comorbidities that are more subjective in nature. Odds ratios for overall agreement for each comorbidity stratified by age, race, marital status, employment status, and education are summarized in Table S2.

**FIGURE 3:**
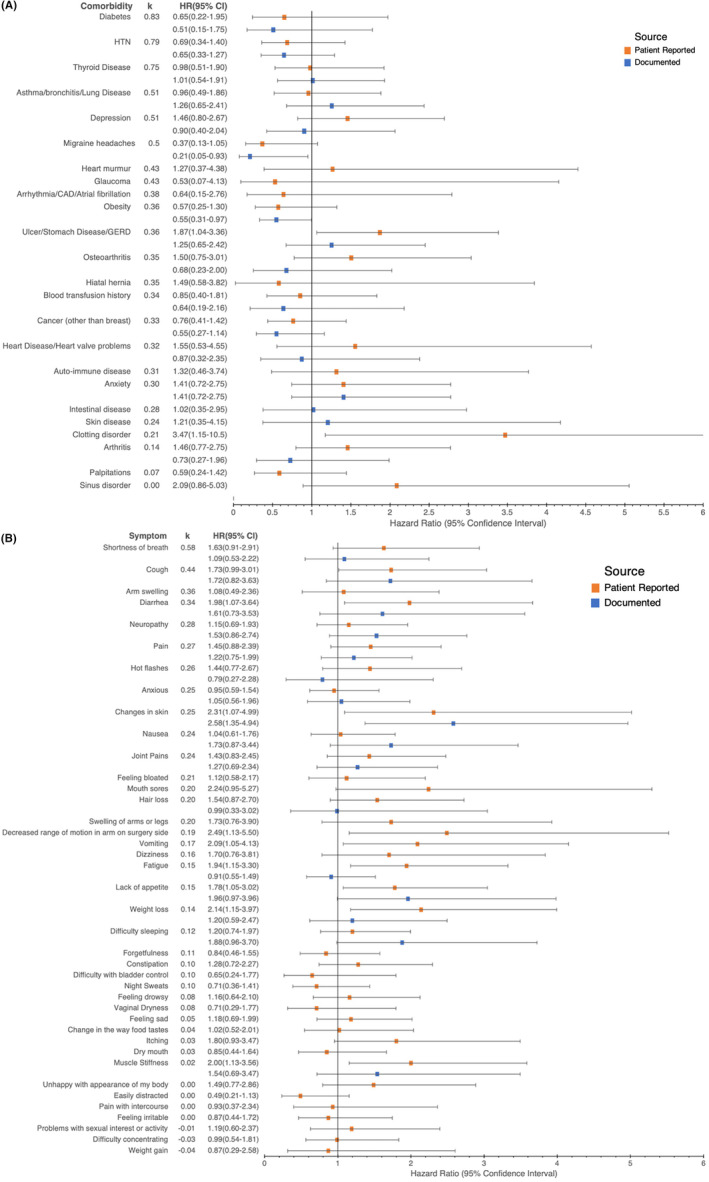
Hazard ratio (HR) associated with patient‐reported and physician‐documented comorbidities (A) and symptoms (B). Error bars represent 95% confidence intervals. Comorbidities (in A) and symptoms (in B) are ordered by κ.

Hazard ratios (HR) generated through cox‐proportional hazards for each comorbidity after controlling for clinical covariates are summarized in Figure 3A. Patient‐reported stomach ulcers/GERD and clotting disorders were associated significantly with lower survival, and physician‐documented migraine disease was significantly associated with increased survival (HR = 1.87, 3.47 and 0.21 respectively, *p* < 0.05).

### 3.3 Symptom analyses

Patient and physician reporting of each symptom, level of agreement, and *κ* are summarized in Figure 2B and Table S3. Pain, lack of energy, and anxiety were the most highly documented and reported symptoms by both physicians and patients. With the exception of changes in skin, patients reported higher percentages of a symptom than physicians documented. Concordance between patient and physician reports of symptoms was poor for most symptoms. Only 2 of 42 symptoms had a moderate to high level of agreement between patients and physicians (*κ* ≥ 0.40 for shortness of breath and cough).

The only gastrointestinal symptoms for which there was even fair concordance were diarrhea and nausea (*κ* = 0.34 and 0.24 respectively), while concordance for vomiting and constipation was poor (*κ* = 0.17 and 0.10 respectively). The only endocrinological symptom or symptom related to sexual function with fair concordance was hot flashes/flushes (*κ* = 0.26), while concordance was poor for pain with intercourse, vaginal dryness, problems with sexual interest, and night sweats (*κ* = 0.00, 0.08, −0.01, and 0.10, respectively). Among symptoms that are included in the Edmonton Symptom Assessment Scale (ESAS), among this patient population, shortness of breath has a moderate level of agreement (*κ* = 0.58), while pain, nausea, and anxiety had fair agreement (*κ* = 0.27, 0.24, and 0.25, respectively). The remaining symptoms had poor agreement between patients and physicians (*κ* = 0.15, 0.05, 0.08, and 0.15 for lack of energy, feeling sad, feeling drowsy, and lack of appetite, respectively). Odds ratios for overall agreement for each comorbidity stratified by age, race, marital status, employment status, and education are summarized in Table S4.

Hazard ratios (HR) generated through cox‐proportional hazards for each symptom after controlling for clinical covariates are summarized in Figure 3B. Patient‐reported cough, diarrhea, changes in skin, decreased range of motion in arm on surgery side, vomiting, fatigue, lack of appetite, weight loss and muscle stiffness, and physician‐documented changes in skin were significantly associated with decreased survival (HR = 1.73, 1.98, 2.31, 2.49, 2.09, 1.94, 1.78, 2.14, 2.00, and 2.58, respectively, *p* < 0.05).

## 4 DISCUSSION

In this review of data collected from 168 metastatic breast cancer patients as part of routine care at an academic medical center, we examined the concordance between patient reports and physician documentation of 54 comorbidities 42 symptoms. Further, we looked survival models to examine whether patient or physician reports of comorbidities and symptoms were better predictors of survival.

It is important to accurately assess and document a patient's comorbidities since cancer treatment decision‐making is heavily impacted by a patient's baseline comorbidities and comorbidities that develop as a result of prior cancer treatment.^1^, ^12^, ^13^, ^14^, ^15^ Comorbidities can not only impact whether a patient receives more aggressive or conservative anti‐cancer treatment but also impact eligibility for clinical trials and novel therapeutics. Furthermore, along with tumor biology, comorbidities are considered to be an important predictor of prognosis and survival.^16^, ^17^, ^18^ Our study population had lower prevalence of multiple comorbidities than the general US population, though comparable to the population of metastatic breast cancer patients. The Charlson Comorbidity Index assessed for our population was 6.4 ± 0.88, which is within the range of the previously reported value of 6.3 ± 0.6 (or 0.3 ± 0.6 not including metastatic breast cancer in the calculation)^19^ in a larger sample study of metastatic breast cancer patients.

Patient‐reported comorbidity and symptom data may help physicians more comprehensively document conditions such as depression, anxiety, arthritis, and stomach ulcers/GERD, which may be subjective in nature but can significantly impact a patient's quality of life and performance status. While, ideally, patient‐reported subjective comorbidities should largely overlap and agree with their physician's documentation, this may not be the case practically. While providing history in the examination room to their physician, the patient may forget or not have time to share information about these comorbidities, or may prioritize communicating other information such prior treatments, cancer history, and other comorbidities due to the time constraints and focus on the condition that is most relevant to the visit they are engage in. Additionally, physicians may either not thoroughly document the patient's report or not document all the comorbidities as comprehensively. Additionally, certain extensive physical examinations which might not be necessary for an intake appointment for a patient with cancer may not be performed at this initial appointment. On the contrary, patients may underreport conditions such as obesity and heart disease, and inaccurately report other conditions such as non‐insulin dependent diabetes, which are objectively measured and determined. Additionally, recognition of depression, anxiety, and GERD as comorbidities and improving capture of these conditions by physicians is important since some medications for these conditions may be contraindicated in clinical trials. For example, many proton pump inhibitors are not allowed in trials of many oral anti‐cancer therapies since the change in stomach acidity impacts absorption. Similarly, several antidepressants are contraindicated in trials due to the risk of prolongation of the QT interval.

Overall, patients reported more symptoms than physicians, however, the top three reported symptoms (pain, lack of energy, and anxiety) were the same by both patients and physicians. Concordance between patient and physician reports of symptoms was poor for most symptoms. Previous literature corroborates these results that concordance between patient reports and physician documentation of symptoms is fairly low and medical records usually have lower reports of symptoms,^20^, ^21^ and hence may not provide adequate representation of the symptom burden that patients experience.

Patient‐reported symptoms were also better predictors of patient survival. Interestingly, comorbidities such as diabetes and hypertension did not predict survival in this metastatic population. In our study population, after controlling for clinical covariates of their metastatic breast cancer, most comorbidities (patient‐reported or physician‐documented) were not significant drivers of survival. However, multiple patient‐reported symptoms, including diarrhea, cough, fatigue, weight loss, and changes in skin, were independently associated with reduced survival despite controlling for clinical covariates of the metastatic breast cancer. Our study suggests that in this patient population, the primary drivers of survival were more likely patients’ tumor biology, tumor burden, and patient‐reported symptoms (either related to their comorbidities or their cancer treatment) over the presence of comorbidities that are considered important predictors of survival in the broader population.

Patients report symptoms earlier and more frequently than physicians,^22^ and this seems to suggest that physicians are documenting the more acute or longer lasting symptoms. Thus, we would expect physician reported symptoms to be stronger predictors of survival. However, compared to patient‐reported symptoms, physician‐documented symptoms were not significant predictors of survival. One potential explanation for this might be that symptoms that are being documented by physicians are also those being addressed better by supportive care and therefore having less impact on patient function and quality of life. Patient‐reported symptoms, either secondary to their cancer or treatment or related to their comorbidities, may provide more insight into those symptoms that remain untreated and are more problematic for the patient. Additionally patient‐reported symptoms may provide valuable information to estimate patients' performance status thereby influencing eligibility for clinical trials and assessing the tolerability of standard and novel therapeutics.

There is a current move toward increasing use of patient‐reported outcomes in oncology, since several studies have suggested that incorporating patient‐reported outcomes in cancer care improves patient outcomes, quality of life, and patient satisfaction^23^ and also provide a less expensive option for obtaining patients' health histories.^24^ While several studies have extolled the benefits of incorporating patient‐reported outcomes, there is still a need to establish the most accurate source for documenting patients' true comorbidities and other details of their health history. Our research suggests that for comorbidities, data sourced from patients and physicians are complementary. While physician documentation provides better capture of objectively assessed comorbidities such as obesity, hypertension, diabetes, and thyroid disease, incorporating patient‐reported comorbidities into this data may provide a better representation of comorbidities that cannot be objectively assessed through laboratory tests, imaging, tests, etc. By combining these complementary sources of comorbidities, we may get closer to a more accurate representation of a patient's true health history. However, with respect to capture of symptoms, patient reports appear to be a richer source of data, irrespective of the type of symptom assessed. Patient‐reported symptoms provide not only better capture but also appear to be a better surrogate for evaluating the severity of patients' metastatic disease burden compared to physician‐documented symptoms, as suggested by the stronger association with survival. However, we must note that we did not examine which source of comorbidities or symptoms (patient report or physician‐documented) were accurate when patient report and physician documentation differed.

Though the source for patient‐reported data was an electronic intake survey, which has several benefits, including higher quality data, lower costs, and ability to complete at a self‐preferred pace, there is the potential of reduced representation of certain populations, such as older patients and those with lower technological literacy. Additionally, as an academic medical center, we attract a higher risk and younger patient population, as well as patients with higher education level and health literacy, and who are fit enough to travel for their cancer care.

## 5 CONCLUSION

In our study of this population‐based cohort of new patients with a diagnosis of metastatic breast cancer at an academic medical center, we found that agreement between patients and physicians in reporting of comorbidities has substantial variance, and patient‐reported comorbidities may complement physician‐documented comorbidities to provide a more comprehensive assessment of a patient's medical history. However, physicians significantly underreported symptoms, compared to patients, and, thus, concordance was also low. However, multiple patient‐reported symptoms were significantly predictive of patient survival in this population, even after controlling for patients' cancer biology, suggesting that incorporating patient‐reported symptoms, either secondary to their cancer or related to their comorbidities, may provide a more informative estimate of a patients' predicted survival, and assist physicians in evaluating trial eligibility and reasonable treatment options.

## AUTHOR CONTRIBUTIONS

**Saumya Umashankar:** Conceptualization (equal); data curation (equal); formal analysis (lead); investigation (lead); methodology (equal); software (lead); validation (lead); visualization (lead); writing – original draft (equal); writing – review and editing (equal). **Amrita Basu:** Conceptualization (equal); formal analysis (equal); investigation (equal); methodology (equal); supervision (lead); validation (equal); visualization (equal); writing – original draft (equal); writing – review and editing (equal). **Laura J. van't Veer:** Funding acquisition (equal); project administration (equal); resources (equal); supervision (equal); writing – review and editing (equal). **Laura J. Esserman:** Funding acquisition (equal); project administration (equal); resources (equal); supervision (equal); writing – review and editing (equal). **Michelle Melisko:** Conceptualization (equal); data curation (lead); investigation (equal); methodology (equal); resources (lead); supervision (lead); visualization (equal); writing – original draft (equal); writing – review and editing (equal).

## FUNDING INFORMATION

This study was funded by the Safeway Foundation, Give Breast Cancer the Boot, and the University of California Office of the President, A114103.

## CONFLICT OF INTEREST STATEMENT

The authors declare no conflicts of interest.

## Supporting information


Tables S1–S4.

## Data Availability

Data available on request from the authors.

## Footnotes

* Race was a multiple‐option checkbox, and patients could select more than one of the options to best describe their self‐identified race.
