# Supplementary material for: Concordance between patient‐reported and physician‐documented comorbidities and symptoms among Stage 4 breast cancer patients
Source: Cancer Med. 2023 Oct 30;12(22):20906–17. doi: 10.1002/cam4.6632 (PMC10709717; doi:10.1002/cam4.6632)
Supplement: Supplementary file 1 — Tables S1–S4. [file CAM4-12-20906-s001.docx]

Supplementary Table 1: Concordance between patient reported and physician documented comorbidities

| *Comorbidity* | **Physician Documented** | **Patient Reported** | **Concordant** | **Cohen's Kappa (κ)** |
| --- | --- | --- | --- | --- |
| *Bleeding disorders* | 1% | 1% | 100% | 1 |
| *GI Bleed* | 1% | 1% | 100% | 1 |
| *Pulmonary embolism* | 1% | 1% | 100% | 1 |
| *Diabetes* | 6% | 5% | 98% | 0.83 |
| *High Blood Pressure* | 24% | 20% | 93% | 0.79 |
| *Thyroid Disease* | 20% | 18% | 92% | 0.75 |
| *Chest pain* | 1% | 1% | 99% | 0.66 |
| *Diabetes Mellitus - IDDM* | 1% | 1% | 99% | 0.66 |
| *Heart Attack* | 1% | 1% | 99% | 0.66 |
| *Stroke* | 1% | 1% | 99% | 0.66 |
| *Substance abuse* | 1% | 1% | 99% | 0.66 |
| *Hepatitis chronic* | 2% | 1% | 99% | 0.66 |
| *Melanoma* | 1% | 2% | 99% | 0.66 |
| *Deep Vein Thrombosis (DVT)* | 2% | 2% | 98% | 0.56 |
| *Asthma/bronchitis/Lung Disease* | 12% | 15% | 89% | 0.51 |
| *Depression* | 11% | 20% | 87% | 0.51 |
| *Migraine headaches* | 8% | 13% | 90% | 0.5 |
| *Blood disorder* | 1% | 1% | 99% | 0.49 |
| *Liver Disease* | 2% | 3% | 98% | 0.49 |
| *Kidney Disease* | 2% | 2% | 98% | 0.49 |
| *Heart murmur* | 1% | 4% | 97% | 0.43 |
| *Glaucoma* | 2% | 4% | 97% | 0.43 |
| *Congestive Heart Failure* | 2% | 1% | 98% | 0.39 |
| *Arrhythmias/Coronary Artery Disease/ Atrial fibrillation* | 2% | 4% | 96% | 0.38 |
| *Obesity/Morbid Obesity* | 34% | 15% | 75% | 0.36 |
| *Stomach Ulcers/GERD* | 11% | 18% | 84% | 0.36 |
| *Osteoarthritis* | 8% | 13% | 88% | 0.35 |
| *Hiatal hernia* | 2% | 5% | 96% | 0.35 |
| *Blood transfusion in the past* | 4% | 11% | 90% | 0.34 |
| *Cancer (other than breast cancer)* | 11% | 18% | 83% | 0.33 |
| *Psychiatric treatment (mental illness)* | 1% | 2% | 98% | 0.32 |
| *Heart Disease/ Heart valve problems* | 7% | 4% | 93% | 0.32 |
| *Auto-immune disease* | 2% | 5% | 95% | 0.31 |
| *Anxiety or Panic Attacks* | 10% | 18% | 83% | 0.3 |
| *Intestinal disease/ problems* | 5% | 3% | 95% | 0.28 |
| *Diabetes Mellitus - NDDM* | 3% | 1% | 97% | 0.27 |
| *Skin disease* | 4% | 1% | 96% | 0.24 |
| *Clotting disorder* | 1% | 4% | 96% | 0.21 |
| *Arthritis* | 9% | 20% | 78% | 0.14 |
| *Palpitations* | 2% | 11% | 89% | 0.07 |
| *Alzheimer’s disease, dementia* | 1% | 0% | 99% | 0 |
| *Peripheral vascular disease* | 0% | 1% | 99% | 0 |
| *Seizures/ epilepsy* | 1% | 0% | 99% | 0 |
| *Tuberculosis (TB)* | 0% | 1% | 99% | 0 |
| *Immune disorders* | 0% | 3% | 97% | 0 |
| *Sinus disorder* | 0% | 5% | 95% | 0 |
| *Emphysema/ COPD* | 1% | 1% | 99% | -0.01 |
| *Nerve/muscle disease* | 1% | 1% | 99% | -0.01 |
| *Ulcers* | 1% | 2% | 98% | -0.01 |
| *Angina* | 0% | 0% | 100% |  |
| *Asbestos exposure* | 0% | 0% | 100% |  |
| *Cirrhosis (liver failure)* | 0% | 0% | 100% |  |
| *HIV/AIDS* | 0% | 0% | 100% |  |
| *Pancreatitis (chronic)* | 0% | 0% | 100% |  |
| *Rheumatoid arthritis* | 1% | 2% | 98% |  |

Supplementary Table 2: Odds ratios for overall agreement for comorbidities stratified by demographics

|  | Age | | Education | | Employment | | Marital Status | | Race | |
| --- | --- | --- | --- | --- | --- | --- | --- | --- | --- | --- |
| *Comorbid Condition* | **Age <60** | **Age > 60** | **Some college or less** | **College graduate or more** | **Not employed** | **Employed** | **Not Married** | **Married** | **White** | **Non-white** |
| *Obesity* | 1 | 0.45 | 1 | 0.66 | 1 | 1.56 | 1 | 1.84 | 1 | 0.76 |
|  | Ref | (0.2-1) | Ref | (0.31-1.37) | Ref | (0.75-3.23) | Ref | (0.71-4.8) | Ref | (0.3-1.91) |
| *Cancer (other than breast)* | 1 | 3.19 | 1 | 0.99 | 1 | 0.37 | 1 | 0.51 | 1 | 0.78 |
|  | Ref | (1.38-7.38) | Ref | (0.41-2.36) | Ref | (0.13-1.04) | Ref | (0.21-1.26) | Ref | (0.27-2.24) |
| *Lung Disease/ Asthma/ bronchitis* | 1 | 1.25 | 1 | 1.36 | 1 | 0.67 | 1 | 0.56 | 1 | 0.44 |
|  | Ref | (0.47-3.31) | Ref | (0.46-3.99) | Ref | (0.23-1.97) | Ref | (0.19-1.58) | Ref | (0.1-2.04) |
| *Arthritis* | 1 | 4.13 | 1 | 0.92 | 1 | 0.59 | 1 | 0.65 | 1 | 0.41 |
|  | Ref | (1.9-8.98) | Ref | (0.42-2.02) | Ref | (0.26-1.36) | Ref | (0.28-1.52) | Ref | (0.13-1.25) |
| *Osteoarthritis* | 1 | 6.32 | 1 | 0.53 | 1 | 0.31 | 1 | 0.36 | 1 | 0.88 |
|  | Ref | (2.17-18.42) | Ref | (0.2-1.36) | Ref | (0.09-1.11) | Ref | (0.13-0.95) | Ref | (0.27-2.82) |
| *Thyroid Disease* | 1 | 2.93 | 1 | 6.18 | 1 | 0.57 | 1 | 3.56 | 1 | 0.63 |
|  | Ref | (0.91-9.41) | Ref | (0.78-48.83) | Ref | (0.15-2.15) | Ref | (0.45-28.34) | Ref | (0.13-2.98) |
| *Auto-immune disease* | 1 | 1.72 | 1 | 0.77 | 1 | 0.64 | 1 | 0.44 | 1 | 2.25 |
|  | Ref | (0.42-7.16) | Ref | (0.18-3.36) | Ref | (0.13-3.29) | Ref | (0.1-1.94) | Ref | (0.51-9.92) |
| *Migraine headaches* | 1 | 0.74 | 1 | 1.46 | 1 | 2.12 | 1 | 0.57 | 1 | 0.22 |
|  | Ref | (0.25-2.24) | Ref | (0.45-4.75) | Ref | (0.75-6) | Ref | (0.19-1.77) | Ref | (0.03-1.69) |
| *Heart Disease* | 1 | 2.12 | 1 | 2.21 | 1 | 0.42 | 1 | 0.3 | 1 | 1.37 |
|  | Ref | (0.62-7.27) | Ref | (0.46-10.59) | Ref | (0.09-1.99) | Ref | (0.09-1.05) | Ref | (0.34-5.47) |
| *High Blood Pressure* | 1 | 1.22 | 1 | 2.48 | 1 | 0.37 | 1 | 0.52 | 1 | 1.37 |
|  | Ref | (0.37-4.01) | Ref | (0.52-11.72) | Ref | (0.08-1.75) | Ref | (0.15-1.85) | Ref | (0.34-5.47) |
| *Heart murmur* | 1 | 7.1 | 1 | 1.91 | 1 | 0.48 | 1 | 0.4 | 1 | 1.2 |
|  | Ref | (0.78-65.06) | Ref | (0.21-17.5) | Ref | (0.05-4.42) | Ref | (0.06-2.5) | Ref | (0.12-11.87) |
| *Palpitations* | 1 | 1.6 | 1 | 1.02 | 1 | 1.17 | 1 | 0.56 | 1 | 1.32 |
|  | Ref | (0.61-4.17) | Ref | (0.36-2.85) | Ref | (0.43-3.15) | Ref | (0.19-1.58) | Ref | (0.44-3.96) |
| *Arrhythmias/ Coronary Artery Disease* | 1 | 0.83 | 1 | 2.41 | 1 | 0.38 | 1 | 0.26 | 1 | 1.83 |
|  | Ref | (0.15-4.69) | Ref | (0.27-21.13) | Ref | (0.04-3.35) | Ref | (0.05-1.35) | Ref | (0.32-10.45) |
| *Stomach Disease/ GERD* | 1 | 2.04 | 1 | 0.52 | 1 | 0.29 | 1 | 1.72 | 1 | 0.44 |
|  | Ref | (0.89-4.69) | Ref | (0.23-1.21) | Ref | (0.1-0.89) | Ref | (0.55-5.34) | Ref | (0.12-1.56) |
| *Diabetes Mellitus - NDDM* | 1 | 1.12 | 1 | 0.11 | 1 | 1.32 | 1 | 1.11 | 1 | 0 |
|  | Ref | (0.18-6.91) | Ref | (0.01-1) | Ref | (0.21-8.14) | Ref | (0.12-10.26) | Ref | (0-NaN) |
| *Depression* | 1 | 0.95 | 1 | 2.32 | 1 | 1.43 | 1 | 0.42 | 1 | 0.56 |
|  | Ref | (0.38-2.42) | Ref | (0.74-7.24) | Ref | (0.57-3.58) | Ref | (0.16-1.11) | Ref | (0.15-2.01) |
| *Anxiety or Panic Attacks* | 1 | 0.4 | 1 | 1.21 | 1 | 0.48 | 1 | 1.02 | 1 | 1.02 |
|  | Ref | (0.15-1.05) | Ref | (0.49-2.96) | Ref | (0.18-1.26) | Ref | (0.38-2.74) | Ref | (0.38-2.77) |
| *Hiatal hernia* | 1 | 4.47 | 1 | 0.61 | 1 | 0.78 | 1 | 0.35 | 1 | 0.71 |
|  | Ref | (0.84-23.8) | Ref | (0.13-2.84) | Ref | (0.15-4.14) | Ref | (0.07-1.64) | Ref | (0.08-6.25) |
| *Immune disorders* | 1 | 2.59 | 1 | 0.7 | 1 | 0.48 | 1 | 0.4 | 1 | 2.46 |
|  | Ref | (0.42-15.97) | Ref | (0.11-4.29) | Ref | (0.05-4.42) | Ref | (0.06-2.5) | Ref | (0.4-15.37) |
| *Intestinal disease* | 1 | 2.19 | 1 | 0.93 | 1 | 0.54 | 1 | 2.3 | 1 | 0.5 |
|  | Ref | (0.57-8.5) | Ref | (0.22-3.89) | Ref | (0.11-2.71) | Ref | (0.28-18.98) | Ref | (0.06-4.17) |
| *Glaucoma* | 1 | 2.59 | 1 | 0.7 | 1 | 0.48 | 1 | 0.4 | 1 | 5.77 |
|  | Ref | (0.42-15.97) | Ref | (0.11-4.29) | Ref | (0.05-4.42) | Ref | (0.06-2.5) | Ref | (0.92-35.98) |
| *Sinus disorder* | 1 | 2.95 | 1 | 3.43 | 1 | 0.64 | 1 | 0.82 | 1 | 0 |
|  | Ref | (0.68-12.81) | Ref | (0.41-28.65) | Ref | (0.13-3.29) | Ref | (0.16-4.26) | Ref | (0-NaN) |
| *Skin disease* | 1 | 3.52 | 1 | 0.94 | 1 | 0.38 | 1 | 1.4 | 1 | 0 |
|  | Ref | (0.62-19.79) | Ref | (0.17-5.28) | Ref | (0.04-3.35) | Ref | (0.16-12.38) | Ref | (0-NaN) |
| *Blood transfusion in the past* | 1 | 1.34 | 1 | 0.76 | 1 | 0.88 | 1 | 2.05 | 1 | 0.48 |
|  | Ref | (0.47-3.81) | Ref | (0.26-2.22) | Ref | (0.29-2.68) | Ref | (0.44-9.48) | Ref | (0.1-2.22) |
| *Clotting disorder* | 1 | 2.32 | 1 | 1.18 | 1 | 0.32 | 1 | 1.69 | 1 | 1.45 |
|  | Ref | (0.5-10.74) | Ref | (0.22-6.29) | Ref | (0.04-2.68) | Ref | (0.2-14.54) | Ref | (0.27-7.84) |

Supplementary Table 3: Concordance between patient reported and physician documented symptoms

| *Symptoms* | **Physician Documented** | **Patient Reported** | **Concordant** | **Cohen's Kappa (κ)** |
| --- | --- | --- | --- | --- |
| *Shortness of breath* | 12% | 20% | 89% | 0.58 |
| *Cough* | 14% | 22% | 83% | 0.44 |
| *Arm swelling* | 2% | 10% | 92% | 0.36 |
| *Diarrhea* | 11% | 20% | 83% | 0.34 |
| *Problems with urination* | 4% | 4% | 95% | 0.28 |
| *Numbness/tingling in hands/feet* | 19% | 34% | 71% | 0.28 |
| *Pain* | 48% | 53% | 63% | 0.27 |
| *Hot flashes or flushes* | 9% | 22% | 80% | 0.26 |
| *Changes in skin* | 13% | 12% | 84% | 0.25 |
| *Anxious* | 20% | 47% | 64% | 0.25 |
| *Nausea* | 9% | 26% | 77% | 0.24 |
| *Joint Pains* | 17% | 28% | 73% | 0.24 |
| *Feeling bloated* | 5% | 17% | 84% | 0.21 |
| *Mouth sores* | 3% | 7% | 92% | 0.2 |
| *Swelling of arms or legs* | 5% | 9% | 89% | 0.2 |
| *Hair loss* | 7% | 19% | 81% | 0.2 |
| *Decreased range of motion in arm on surgery side* | 2% | 10% | 91% | 0.19 |
| *Vomiting* | 2% | 10% | 90% | 0.17 |
| *Dizziness* | 1% | 7% | 94% | 0.16 |
| *Lack of appetite* | 9% | 27% | 73% | 0.15 |
| *Lack of energy* | 45% | 55% | 57% | 0.15 |
| *Weight loss* | 13% | 14% | 80% | 0.14 |
| *Difficulty sleeping* | 13% | 36% | 65% | 0.12 |
| *Forgetfulness* | 4% | 24% | 77% | 0.11 |
| *Difficulty with bladder control* | 2% | 8% | 92% | 0.1 |
| *Night Sweats* | 2% | 17% | 83% | 0.1 |
| *Constipation* | 5% | 21% | 78% | 0.1 |
| *Vaginal Dryness* | 2% | 10% | 90% | 0.08 |
| *Feeling drowsy* | 2% | 19% | 81% | 0.08 |
| *Feeling sad* | 5% | 31% | 69% | 0.05 |
| *Change in the way food tastes* | 2% | 16% | 84% | 0.04 |
| *Itching* | 2% | 14% | 84% | 0.03 |
| *Dry mouth* | 2% | 17% | 82% | 0.03 |
| *Muscle Stiffness* | 10% | 22% | 73% | 0.02 |
| *Pain with intercourse* | 0% | 8% | 92% | 0 |
| *Easily distracted* | 0% | 11% | 89% | 0 |
| *Unhappy with appearance of my body* | 0% | 14% | 86% | 0 |
| *Feeling irritable* | 0% | 16% | 84% | 0 |
| *Problems with sexual interest or activity* | 1% | 14% | 85% | -0.01 |
| *Difficulty swallowing* | 1% | 5% | 94% | -0.02 |
| *Difficulty concentrating* | 2% | 23% | 75% | -0.03 |
| *Weight gain* | 2% | 7% | 90% | -0.04 |

Supplementary Table 4: Odds ratios for overall agreement for symptoms stratified by demographics

|  | Age | | Education | | Employment | | Marital Status | | Race | |
| --- | --- | --- | --- | --- | --- | --- | --- | --- | --- | --- |
| *Comorbid Condition* | **Age <60** | **Age > 60** | **Some college or less** | **College graduate or more** | **Not employed** | **Employed** | **Not Married** | **Married** | **White** | **Non-white** |
| *Difficulty concentrating* | 1 | 0.43 | 1 | 1.23 | 1 | 0.84 | 1 | 0.71 | 1 | 1.21 |
|  | Ref | (0.2-0.96) | Ref | (0.57-2.66) | Ref | (0.4-1.79) | Ref | (0.31-1.61) | Ref | (0.52-2.8) |
| *Lack of energy* | 1 | 1.17 | 1 | 0.96 | 1 | 1.25 | 1 | 0.45 | 1 | 1.42 |
|  | Ref | (0.62-2.2) | Ref | (0.5-1.86) | Ref | (0.65-2.39) | Ref | (0.21-0.95) | Ref | (0.67-3.01) |
| *Cough* | 1 | 1.88 | 1 | 0.81 | 1 | 0.61 | 1 | 1.81 | 1 | 0.74 |
|  | Ref | (0.83-4.25) | Ref | (0.35-1.91) | Ref | (0.24-1.52) | Ref | (0.59-5.61) | Ref | (0.26-2.11) |
| *Dry mouth* | 1 | 0.81 | 1 | 0.65 | 1 | 0.54 | 1 | 1.13 | 1 | 1.56 |
|  | Ref | (0.35-1.86) | Ref | (0.29-1.47) | Ref | (0.22-1.35) | Ref | (0.42-3.02) | Ref | (0.62-3.91) |
| *Nausea* | 1 | 0.44 | 1 | 0.56 | 1 | 0.64 | 1 | 1.3 | 1 | 0.94 |
|  | Ref | (0.19-1) | Ref | (0.26-1.18) | Ref | (0.28-1.43) | Ref | (0.52-3.25) | Ref | (0.39-2.28) |
| *Feeling drowsy* | 1 | 0.91 | 1 | 0.82 | 1 | 0.63 | 1 | 2.1 | 1 | 1.05 |
|  | Ref | (0.4-2.04) | Ref | (0.36-1.87) | Ref | (0.26-1.52) | Ref | (0.68-6.44) | Ref | (0.41-2.69) |
| *Numbness/ tingling in hands/feet* | 1 | 1.01 | 1 | 0.7 | 1 | 0.98 | 1 | 1.29 | 1 | 0.76 |
|  | Ref | (0.5-2.02) | Ref | (0.35-1.42) | Ref | (0.48-1.98) | Ref | (0.55-2.99) | Ref | (0.33-1.78) |
| *Difficulty sleeping* | 1 | 0.51 | 1 | 0.74 | 1 | 1.33 | 1 | 0.51 | 1 | 2.72 |
|  | Ref | (0.26-1.02) | Ref | (0.38-1.46) | Ref | (0.68-2.59) | Ref | (0.24-1.09) | Ref | (1.26-5.85) |
| *Feeling bloated* | 1 | 2.04 | 1 | 0.93 | 1 | 0.39 | 1 | 0.48 | 1 | 1.09 |
|  | Ref | (0.89-4.69) | Ref | (0.39-2.23) | Ref | (0.14-1.1) | Ref | (0.2-1.19) | Ref | (0.4-2.95) |
| *Problems with urination* | 1 | 2.19 | 1 | 0.93 | 1 | 0.98 | 1 | 2.3 | 1 | 0.43 |
|  | Ref | (0.57-8.5) | Ref | (0.22-3.89) | Ref | (0.24-4.08) | Ref | (0.28-18.98) | Ref | (0.05-3.56) |
| *Vomiting* | 1 | 0.21 | 1 | 0.57 | 1 | 1.2 | 1 | Inf | 1 | 1.73 |
|  | Ref | (0.05-0.98) | Ref | (0.2-1.62) | Ref | (0.41-3.49) | Ref | (NaN-Inf) | Ref | (0.56-5.35) |
| *Shortness of breath* | 1 | 1.6 | 1 | 0.29 | 1 | 0.2 | 1 | 0.56 | 1 | 1.78 |
|  | Ref | (0.61-4.17) | Ref | (0.11-0.77) | Ref | (0.05-0.91) | Ref | (0.19-1.58) | Ref | (0.62-5.09) |
| *Diarrhea* | 1 | 0.59 | 1 | 0.51 | 1 | 1.04 | 1 | 0.84 | 1 | 1.47 |
|  | Ref | (0.24-1.42) | Ref | (0.22-1.15) | Ref | (0.45-2.42) | Ref | (0.33-2.17) | Ref | (0.59-3.67) |
| *Feeling sad* | 1 | 0.58 | 1 | 0.74 | 1 | 0.82 | 1 | 1.79 | 1 | 1.19 |
|  | Ref | (0.29-1.17) | Ref | (0.37-1.48) | Ref | (0.41-1.66) | Ref | (0.75-4.26) | Ref | (0.54-2.65) |
| *Night Sweats* | 1 | 1.1 | 1 | 1.89 | 1 | 1.34 | 1 | 0.51 | 1 | 2.2 |
|  | Ref | (0.48-2.54) | Ref | (0.72-4.99) | Ref | (0.58-3.09) | Ref | (0.21-1.26) | Ref | (0.88-5.49) |
| *Anxious* | 1 | 1.19 | 1 | 0.71 | 1 | 0.97 | 1 | 1.62 | 1 | 0.62 |
|  | Ref | (0.62-2.29) | Ref | (0.36-1.4) | Ref | (0.5-1.9) | Ref | (0.72-3.65) | Ref | (0.27-1.4) |
| *Problems with sexual interest or activity* | 1 | 0.48 | 1 | 1 | 1 | 1.12 | 1 | 3.65 | 1 | 0.64 |
|  | Ref | (0.18-1.27) | Ref | (0.4-2.48) | Ref | (0.46-2.74) | Ref | (0.82-16.3) | Ref | (0.2-2) |
| *Itching* | 1 | 0.71 | 1 | 0.48 | 1 | 0.42 | 1 | 1.19 | 1 | 1.39 |
|  | Ref | (0.29-1.74) | Ref | (0.21-1.13) | Ref | (0.15-1.17) | Ref | (0.42-3.43) | Ref | (0.53-3.65) |
| *Lack of appetite* | 1 | 0.33 | 1 | 0.76 | 1 | 0.77 | 1 | 0.92 | 1 | 1.79 |
|  | Ref | (0.15-0.76) | Ref | (0.37-1.57) | Ref | (0.37-1.63) | Ref | (0.4-2.11) | Ref | (0.81-3.97) |
| *Dizziness* | 1 | 1.74 | 1 | 0.1 | 1 | 0 | 1 | 2.6 | 1 | 1.02 |
|  | Ref | (0.48-6.26) | Ref | (0.02-0.5) | Ref | (0-NaN) | Ref | (0.32-21.26) | Ref | (0.2-5.15) |
| *Difficulty swallowing* | 1 | 0.4 | 1 | 0.69 | 1 | 0.83 | 1 | 2.6 | 1 | 0.43 |
|  | Ref | (0.08-1.95) | Ref | (0.19-2.54) | Ref | (0.21-3.35) | Ref | (0.32-21.26) | Ref | (0.05-3.56) |
| *Feeling irritable* | 1 | 0.66 | 1 | 0.76 | 1 | 0.8 | 1 | 1.72 | 1 | 1.31 |
|  | Ref | (0.27-1.62) | Ref | (0.32-1.8) | Ref | (0.33-1.96) | Ref | (0.55-5.34) | Ref | (0.5-3.42) |
| *Hot flashes or flushes* | 1 | 0.57 | 1 | 1.32 | 1 | 2.19 | 1 | 0.83 | 1 | 1.77 |
|  | Ref | (0.24-1.31) | Ref | (0.57-3.08) | Ref | (1.01-4.76) | Ref | (0.34-2.05) | Ref | (0.75-4.2) |
| *Vaginal Dryness* | 1 | 0.2 | 1 | 0.84 | 1 | 3.2 | 1 | 1.33 | 1 | 0.74 |
|  | Ref | (0.04-0.9) | Ref | (0.29-2.42) | Ref | (1.15-8.93) | Ref | (0.36-4.9) | Ref | (0.2-2.75) |
| *Pain with intercourse* | 1 | 0.28 | 1 | 1.62 | 1 | 1.77 | 1 | Inf | 1 | 0.28 |
|  | Ref | (0.06-1.32) | Ref | (0.43-6.14) | Ref | (0.56-5.53) | Ref | (NaN-Inf) | Ref | (0.03-2.21) |
| *Joint Pains* | 1 | 1.17 | 1 | 0.7 | 1 | 0.39 | 1 | 0.68 | 1 | 0.93 |
|  | Ref | (0.58-2.35) | Ref | (0.34-1.43) | Ref | (0.17-0.89) | Ref | (0.31-1.51) | Ref | (0.39-2.18) |
| *Muscle Stiffness* | 1 | 1.08 | 1 | 0.76 | 1 | 0.66 | 1 | 0.92 | 1 | 0.93 |
|  | Ref | (0.53-2.19) | Ref | (0.37-1.57) | Ref | (0.31-1.42) | Ref | (0.4-2.11) | Ref | (0.39-2.18) |
| *Weight gain* | 1 | 0.53 | 1 | 1.46 | 1 | 1.2 | 1 | 0.57 | 1 | 0.88 |
|  | Ref | (0.16-1.72) | Ref | (0.45-4.75) | Ref | (0.41-3.49) | Ref | (0.19-1.77) | Ref | (0.23-3.32) |
| *Forgetfulness* | 1 | 0.68 | 1 | 0.68 | 1 | 1.13 | 1 | 0.75 | 1 | 1.99 |
|  | Ref | (0.32-1.47) | Ref | (0.32-1.44) | Ref | (0.53-2.4) | Ref | (0.32-1.73) | Ref | (0.87-4.51) |
| *Easily distracted* | 1 | 0.44 | 1 | 0.71 | 1 | 1.29 | 1 | 2.39 | 1 | 1.44 |
|  | Ref | (0.14-1.41) | Ref | (0.26-1.94) | Ref | (0.47-3.52) | Ref | (0.52-10.9) | Ref | (0.47-4.35) |
| *Arm swelling* | 1 | 0.48 | 1 | 0.52 | 1 | 1.25 | 1 | 0.6 | 1 | 3.49 |
|  | Ref | (0.13-1.81) | Ref | (0.16-1.62) | Ref | (0.39-4.01) | Ref | (0.17-2.06) | Ref | (1.09-11.17) |
| *Decreased range of motion in arm on surgery side* | 1 | 0.82 | 1 | 0.93 | 1 | 0.69 | 1 | 1.12 | 1 | 0.88 |
|  | Ref | (0.27-2.53) | Ref | (0.3-2.88) | Ref | (0.21-2.28) | Ref | (0.3-4.2) | Ref | (0.23-3.32) |
| *Difficulty with bladder control* | 1 | 1.76 | 1 | 0.43 | 1 | 0.3 | 1 | 1.73 | 1 | 0.3 |
|  | Ref | (0.59-5.29) | Ref | (0.14-1.31) | Ref | (0.07-1.4) | Ref | (0.37-8.1) | Ref | (0.04-2.45) |
| *Mouth sores* | 1 | 1.05 | 1 | 0.37 | 1 | 0.15 | 1 | 0.92 | 1 | 2.81 |
|  | Ref | (0.33-3.37) | Ref | (0.12-1.16) | Ref | (0.02-1.17) | Ref | (0.24-3.52) | Ref | (0.83-9.47) |
| *Change in the way food tastes* | 1 | 0.98 | 1 | 0.44 | 1 | 0.51 | 1 | 0.75 | 1 | 3.12 |
|  | Ref | (0.42-2.31) | Ref | (0.19-1.01) | Ref | (0.19-1.34) | Ref | (0.29-1.95) | Ref | (1.29-7.57) |
| *Weight loss* | 1 | 1.05 | 1 | 0.7 | 1 | 1.09 | 1 | 1.37 | 1 | 1.19 |
|  | Ref | (0.48-2.28) | Ref | (0.32-1.54) | Ref | (0.49-2.41) | Ref | (0.52-3.62) | Ref | (0.48-2.92) |
| *Hair loss* | 1 | 0.91 | 1 | 0.58 | 1 | 1.1 | 1 | 1.55 | 1 | 2.1 |
|  | Ref | (0.4-2.04) | Ref | (0.26-1.3) | Ref | (0.49-2.49) | Ref | (0.55-4.38) | Ref | (0.87-5.04) |
| *Constipation* | 1 | 0.4 | 1 | 0.92 | 1 | 0.98 | 1 | 1.94 | 1 | 1.83 |
|  | Ref | (0.17-0.95) | Ref | (0.42-2.02) | Ref | (0.45-2.14) | Ref | (0.7-5.42) | Ref | (0.79-4.24) |
| *Swelling of arms or legs* | 1 | 1.39 | 1 | 0.71 | 1 | 2.15 | 1 | 0.97 | 1 | 1.11 |
|  | Ref | (0.52-3.74) | Ref | (0.26-1.94) | Ref | (0.8-5.76) | Ref | (0.3-3.14) | Ref | (0.34-3.65) |
| *Unhappy with body appearance* | 1 | 0.55 | 1 | 0.56 | 1 | 0.66 | 1 | 1 | 1 | 1.41 |
|  | Ref | (0.2-1.47) | Ref | (0.23-1.37) | Ref | (0.24-1.77) | Ref | (0.34-2.9) | Ref | (0.51-3.92) |
| *Changes in skin* | 1 | 0.81 | 1 | 0.52 | 1 | 1.19 | 1 | 2.49 | 1 | 1.31 |
|  | Ref | (0.34-1.94) | Ref | (0.23-1.21) | Ref | (0.5-2.8) | Ref | (0.7-8.8) | Ref | (0.5-3.42) |
| *Pain* | 1 | 0.88 | 1 | 1.2 | 1 | 0.93 | 1 | 0.94 | 1 | 0.63 |
|  | Ref | (0.43-1.8) | Ref | (0.57-2.5) | Ref | (0.45-1.94) | Ref | (0.4-2.2) | Ref | (0.28-1.42) |
